# Indapamide-induced Severe Hyponatremia in a Middle-aged Male Patient within Two Weeks

**DOI:** 10.7759/cureus.6515

**Published:** 2019-12-30

**Authors:** Phool Iqbal, Bushra K Laswi, Rashid Kazman, Haajra Fatima, Ali Ait Hssain

**Affiliations:** 1 Internal Medicine, Hamad Medical Corporation, Doha, QAT; 2 Internal Medicine, Hamad General Hospital, Doha, QAT; 3 Anesthesiology/Critical Care, Hamad General Hospital, Doha, QAT

**Keywords:** indapamide, severe hyponatremia, middle-aged man, hyponatremia, male, toxicity, complications

## Abstract

Hyponatremia is one of the most common electrolyte abnormalities and is associated with many conditions. It has been reported in patients receiving diuretics as a physiological consequence of the therapy.

We report an unusual case of severe hyponatremia of 100 mmol/L (Normal range (NR): 136-145 mmol/L) in a 54-year-old middle-aged gentleman within two weeks of commencement of Indapamide, a thiazide-like diuretic. After immediate treatment with intravenous 3% hypertonic saline infusion, discontinuation of indapamide, and ruling out other possible causes of hyponatremia, his serum sodium level improved. He was discharged without having any complicated hospital course and was also followed up for a further five months. The aim of our case report is to highlight an important and life-threatening complication associated with the most commonly prescribed drug and to manage it through cautious correction and monitoring of sodium levels.

## Introduction

Hyponatremia is one of the most common electrolyte abnormalities encountered in patients and is defined as a serum sodium level of less than 135 mmol/l [1].

Thiazide diuretics are commonly prescribed in controlling blood pressure and hyponatremia has been reported in patients receiving diuretics as a natural consequence of the therapy. Few case reports and studies have shown that thiazide or thiazide-like diuretics cause profound hyponatremia (110-125 mmol/l) and most of those cases were attributed to thiazide diuretic usage in females, elderly (≥65 years) and with multiple co-morbid conditions [2-5].

## Case presentation

**Figure 1:**
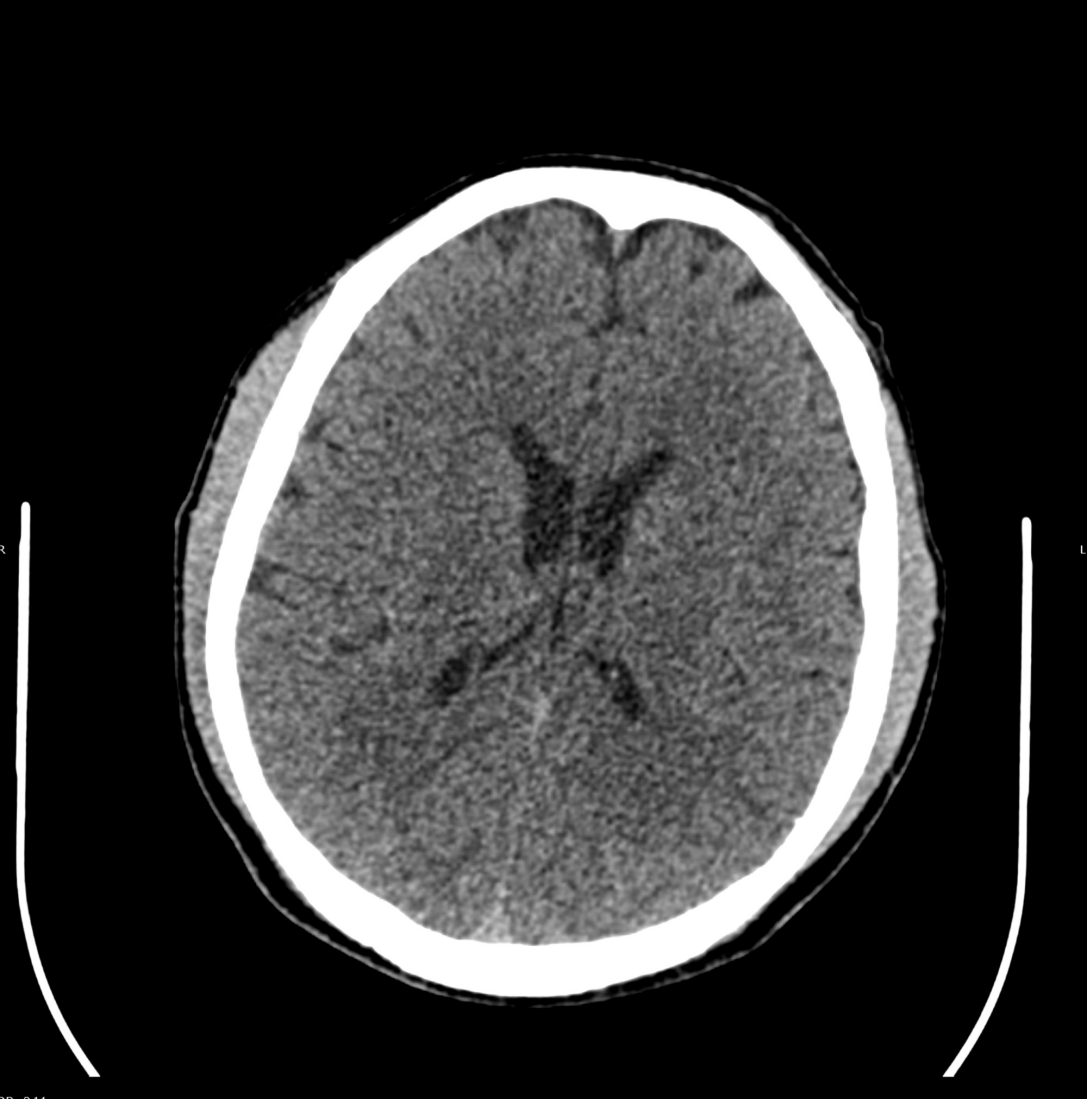
CT scan brain reported normal

A 54-year-old gentleman, known case of hypertension diagnosed during an acute ischemic stroke event without residual weakness, was discharged on anti-hypertensive medications that included perindopril-indapamide (10 mg/2.5 mg). After 10 days of commencement of his medication, he started to have gait imbalance that was aggravated and eventually resulted in a fall but without any loss of consciousness. He sought emergent medical attention. On presentation, he was conscious, alert, oriented and gave a complete history of his presentation. There was no history of vomiting and diarrhea. On examination, he had moist mucous membranes and normal skin turgor without postural hypotension and no signs of overload like raised jugular venous pressure (JVP), peripheral edema or shortness of breath. Later, during his stay in the emergency department, his gait disturbance was increased, associated with dysarthria and he had one episode of generalized tonic-clonic seizures. Due to the history of a recent stroke, an urgent CT scan of the brain was done and found to be unremarkable for any acute brain insult as shown in Figure 1 below.

On blood investigations, his serum sodium level was found to be severely low with a value of 100 mmol/l (NR: 136-145 mmol/L) and a repeat value of 101 mmol/L. Potassium level was also low while the bicarbonate level was high with a value of 2.5 mmol/l (NR: 3.5 mmol/L-5.1 mmol/L) and 34.5 mmol/l (NR: 22-29 mmol/L) respectively. Serum osmolality was 244 mmol/kg (NR: 275-295 mmol/kg) correlating with the hypo-osmolal state, and urine osmolality was in the normal range 342 mosm/kg (NR: 150-1150 mosm/kg). Keeping in mind the most common side effect of thiazide diuretics to cause mild to profound hyponatremia, perindopril-indapamide was withheld.

The patient was started on 3% hypertonic saline infusion for sodium correction. Potassium replacement was given as well. His sodium level along with the rest of the electrolytes also improved and no further drop was observed (Table 1). The patient improved symptomatically over the course of hospital stay without any complications.

**Table 1:** Sodium levels during the course of hospital stay

Laboratory values	At admission	Next day	At discharge
Serum sodium (mmol/l)	100	107	127
Serum potassium (mmol/l)	2.5	2.8	3.8
Serum chloride (mmol/l)	<60	65	89
Serum bicarbonate (mmol/l)	34.5	30	24
Urine osmolarity	342	-	-
Serum osmolarity	244	-	-

## Discussion

Hyponatremia is defined as serum sodium level <135 mmol/l. Joint European guidelines classify hyponatremia as mild (130-134 mmol/l), moderate (125-129 mmol/l) and profound (<125 mmol/l) [1]. Hyponatremia depending upon the serum osmolality can also be classified as normo-osmolal or pseudo-hyponatremia, hypo-osmolal or true hyponatremia and hyperosmolal or translocational hyponatremia with normal serum osmolality (280-295 mosm/kg), low serum osmolality (<280 mosm/kg) and high serum osmolality (>295 mosm/kg), respectively. Further, true hyponatremia (hypo-osmolal) is classified according to the volume status of the patient as hypovolemic, hypervolemic or euvolemic [6]. According to the time of presentation, hyponatremia occurring in less than 48 hours is considered acute and after 48 hours as chronic. When serum sodium levels decrease significantly over a short period of time usually <48 hours, brain edema ensues more frequently as neurons get less time to adapt, and the patient presents with central nervous symptoms ranging from headache, fatigue, nausea, gait disturbance to confusion and even seizures [1].

In our case, the patient had severe chronic hyponatremia (>48 hours) with serum sodium levels of 100 mmol/l (NR: 136 mmol/L-145 mmol/L). He had no co-morbidities associated with any psychiatric illness, fluid overload conditions like heart failure, liver cirrhosis or renal failure and therefore our patient was clinically euvolemic. Laboratory investigations revealed decreased serum osmolality of 244 mmol/kg (NR: 275-295 mmol/kg), normal glucose, proteins, and cholesterol level at presentation, thus further categorizing the patient into true hyponatremia of euvolemic type.

In this category, diuretic-induced hyponatremia, hypothyroidism, syndrome of inappropriate ADH secretion (SIADH) and psychogenic polydipsia were the differentials diagnosis [6]. As our patient was on diuretics, it is difficult to exclude SIADH in such scenarios as diagnostic criteria for SIADH include a diuretic-free period of at least one week [7]. Based on his medical history, clinical findings, and laboratory investigations revealing decreased serum sodium, potassium and chloride levels with high bicarbonate, diuretic-induced hyponatremia was the most likely diagnosis. Moreover, low potassium and high bicarbonate levels are also observed as side effects seen with diuretic use [8, 9].

His thyroid functions revealed low thyroid stimulating hormone (TSH) 0.12 mIU/l (NR: 0.30-4.20 mIU/l) and high free thyroxine (FT4) of 32.1 pmol/l (NR: 11.6-21.9 pmol/l), which showed hyperthyroid state, rather than hypothyroid. His urinary sodium was less than 60 mmol/l and our laboratory equipment calibration did not specify the value of urinary sodium. As mentioned earlier, in relation to his medical history clinical presentation and presence of a triggering factor that is diuretic use, we labeled our patient as diuretic-induced hyponatremia.

Indapamide is a thiazide-like diuretic which lacks the benzothiadiazine core present in thiazide diuretics and exerts a weak diuretic effect by blocking Na/Cl co-transport in the distal convoluted tubules. It causes a decrease in blood pressure by increasing the levels of prostacyclin in the vascular smooth muscle cells and producing vasodilation. It also reduces ventricular hypertrophy relatively more than enalapril and atenolol, and microalbuminuria in diabetic and hypertensive patients, thus making it the most commonly prescribed drug [10].

There are case reports and case studies in the literature that have shown an association of severe hyponatremia with a thiazide or thiazide-like diuretic usage. However, most of it was found and studied in the elderly and female population. Our case highlights the importance of indapamide-induced profound life-threatening hyponatremia in a middle-aged man within two weeks, which has not been reported much in the literature.

In a review of one of the clinical studies done on 223 patients, between January 1994 to April 2002, 70% of the patients having symptomatic hyponatremia with thiazide diuretic usage were females and elderly [2].

A retrospective study analysis on the incidence of osmotic demyelination and mortality with profound hyponatremia has also shown that females and the elderly population were most commonly associated with this complication and the most common etiological factor in this study was thiazide or indapamide usage as compared to SIADH and hypovolemia [5].

In another retrospective case-control study involving 223 cases and 216 controls, it has shown that the increased patient age, immobility, low body mass index (BMI), low serum potassium level and indapamide usage are associated with hyponatremia [11].

In literature, a case has been reported of severe hyponatremia dropping to a level of 99 meq/l in association with thiazide use in an elderly lady of 86 years with minimal symptoms [7].

The body is more prone to have hyponatremia with increasing age and therefore it is commonly seen in the elderly population [2, 3, 7, 9, 11, 12]. Elderly population often have co-morbid conditions like multiple endocrinopathies, hypothyroidism, heart failure, chronic kidney disease, liver failure, hypertension, diabetes mellitus, multiple medications usage like diuretics, non-steroidal anti-inflammatory drugs (NSAIDs), tricyclic anti-depressants (TCAs), tea and toast diet with more water and less protein intake from many years and age-related decrease in glomerular filtration rate (GFR), all of which plays a crucial role in causing low sodium state chronically or even acutely [4, 12]. On the contrary, our case report is unique as it specifically highlights the association of severe hyponatremia with indapamide to the level of 100 meq/l within two weeks of commencement in a middle-aged man without co-morbid illnesses.

The incidence of hyponatremia and hypokalemia in relation to indapamide has been reported with ECG changes presenting as prolongation of QT interval [13, 14]. It highlights the importance of the association between indapamide usage and electrolytes disturbance as in our case and also implies the importance of cautious approach, close monitoring and follow-up while prescribing indapamide [5, 14].

Our patient presented with central nervous symptoms and he was started on 3% hypertonic saline under the medical intensive care unit (MICU) for close observation and monitoring. Indapamide was discontinued and his serum sodium level improved without having any complicated course. The patient was further followed for five months which revealed no recurrent hyponatremia and hospitalization. It has been established in many case reports that the discontinuation of the triggering factor is recommended and it also avoids recurrence [2, 9]. Overcorrection of serum sodium levels >12 mmol/l over 24 hours should be avoided as it can lead to osmotic demyelination syndrome [5]. Therefore extreme caution must be taken in patients treated with indapamide/thiazide especially elderly females while correcting serum sodium levels [2, 5].

## Conclusions

Indapamide is commonly used to control blood pressure and has been shown to be effective for many years. However, its association with severe hyponatremia, in a relatively younger male patient without multiple co-morbid conditions has not been reported much in the literature. Our main goal is to highlight its significance in terms of management when encountered in such scenarios, as it can easily be missed if not identified as the triggering factor. One should be very cautious while correction of sodium with careful monitoring. Discontinuation is mandatory to avoid future recurrence.
